# Web-Based Application to Support Caregivers in the Use of Learning Optimization Methods: Participatory Action Research Study

**DOI:** 10.2196/76543

**Published:** 2026-02-26

**Authors:** Chantal Viscogliosi, Hubert Kenfack Ngankam, Romane Duquette-Laplante, Sarah Rahimaly, Hélène Pigot, Bernard Chassé, Sara Ait Bouziaren, Yves Couturier, Jessica Déry, Nicolas Biard, Jordan Duchesne, Dominique Giroux, Sabrina Jacob, Kim Routhier-Chevrier, Jordan Mino-Roy, Mélanie Caron, Véronique Provencher

**Affiliations:** 1 École de réadaptation, Faculté de médecine et des sciences de la santé, Université de Sherbrooke, 3001, 12e avenue Nord, Sherbrooke, QC, J1H 5N4, Canada, 1 819 821-8000 #72934; 2 Département d'informatique, Faculté des sciences, Université de Sherbrooke, Sherbrooke, QC, Canada; 3 Département de psychologie, Faculté de lettres et sciences humaines, Université de Sherbrooke, Sherbrooke, QC, Canada; 4 Département d'informatique, Faculté des sciences, Université de Sherbrooke, Sherbrooke, QC, Canada; 5 Société Alzheimer des Maskoutains - Vallée des Patriotes, Saint-Hyacinthe, QC, Canada; 6 École de travail social, Faculté des lettres et sciences humaines, Université de Sherbrooke, Sherbrooke, QC, Canada; 7 CIUSSS Mauricie-Centre-du-Québec, Trois-Rivières, QC, Canada; 8 École des sciences de la réadaptation, Université Laval, Québec, QC, Canada; 9 CEGEP de Sherbrooke, Sherbrooke, QC, Canada

**Keywords:** web application, major neurocognitive disorder, family caregivers, intervention, learning-optimizing methods, qualitative research

## Abstract

**Background:**

Major neurocognitive disorders (MNCDs) frequently lead to difficulties in performing activities. Several studies have shown that people living with an MNCD benefit from the use of learning optimization methods from cognitive rehabilitation, such as error-free learning, motor encoding, spaced retrieval, and fading, which promote the safe pursuit of their meaningful activities. However, while the principles of learning optimization methods are relatively straightforward, the personalized application of these methods to the specific situations encountered can be more difficult.

**Objective:**

The aim of this study was to describe the codevelopment process, including the validation of a web application called “Aide-Mémoire-Interactif (AMI),” a tool to help in personalizing learning optimization methods.

**Methods:**

To design the web application, participatory action research based on a codevelopment and validation process was carried out. The various stages leading to the codevelopment of the AMI web application were grouped into 3 phases: (1) assessment of caregivers’ needs (identification of situations frequently encountered by people living with an MNCD); (2) production of the first version of the AMI web application; and (3) validation of the AMI application. Individual interviews and workshops were conducted with 20 participants (caregivers, health and social service professionals, and community organization workers) to obtain a diversity of viewpoints and application contexts of use.

**Results:**

The AMI web application was developed through an iterative, participatory process involving caregivers and professionals. Participants identified daily situations requiring the use of learning optimization methods and provided feedback that guided successive refinements to the content, answer choices, navigation, and personalization features. The logical architecture was built according to the rationale underlying learning optimization methods. The final version enables users to generate tailored strategies for specific situations, access educational capsules, and save personalized strategies. Participants reported improved clarity, usability, and relevance.

**Conclusions:**

This participatory action research enabled the codevelopment and validation of the AMI web application to support the operationalization of learning optimization methods for people living with an MNCD by personalizing them to the situations encountered daily as well as to the underlying cognitive difficulties. By involving caregivers, professionals, and users, this process contributed to the development of a web application that meets user needs and their appreciation.

## Introduction

Major neurocognitive disorders (MNCDs) frequently lead to difficulties in performing activities [1]. These difficulties can be attributed particularly to the deterioration of certain cognitive functions, which hinders the learning of new tasks or the maintenance of known tasks [2]. These difficulties increase as the MNCD progresses, ranging from difficulties in performing complex activities such as managing finances and planning meals to activities of daily living such as hygiene and eating. However, procedural memory and implicit retrieval processes are relatively well preserved compared to other cognitive functions, enabling the continuation of automatic, well-integrated sequences of actions in everyday life. Learning methods that build on these preserved capacities and compensate for the cognitive difficulties arising from MNCDs have been shown to be effective in maintaining the ability to perform activities [2,3].

Several studies have shown that people living with an MNCD benefit from the use of learning optimization methods from cognitive rehabilitation, such as error-free learning, motor encoding, spaced retrieval, and fading, which promote the safe pursuit of their meaningful activities [3-8]. These learning optimization methods provide successful experiences by ensuring that the right challenge is offered [3], thus helping to foster their engagement in the pursuit of meaningful activities and increasing the sense of competence of the caregiver of a person living with an MNCD [7]. These learning optimization methods rely on the cognitive capabilities generally preserved in people living with MNCDs, namely procedural memory and implicit retrieval processes [9]. These methods focus on the automation of correct action sequences during learning (errorless learning and motor encoding [10,11]), the gradually spaced repetition of these sequences (spaced retrieval [12,13]), and the progressive reduction of motor, visual, and verbal cues provided during their performance (vanishing cues [14]). To integrate these learning optimization methods, the involvement of a caregiver or an intervener is necessary to ensure consistency in the sequence of activities and to enable learning [15]. Since the integration of these methods, supported by the caregiver, supports the pursuit of meaningful activities, it fosters a positive relationship within the dyad, which may explain why the introduction of these methods does not increase caregiver burden [16].

However, while the principles of learning optimization methods are relatively straightforward, the personalized application of these methods to specific situations encountered can be more difficult [12,17]. In addition to considering the person’s specific cognitive difficulties (eg, difficulty sequencing tasks, forgetting to eat, or missing appointments), caregivers must also take into account the person’s habits and daily routine [16]. It is therefore essential to offer support or tools to caregivers to help them apply learning optimization methods to the specific situations experienced daily by the person living with an MNCD [17]. Support and tools for caregivers in the personalized application of learning optimization methods could optimize the pursuit of meaningful activities by people living with an MNCD by promoting the use of their preserved abilities.

Many support resources and tools are available for caregivers of people living with MNCDs. According to a recent comprehensive meta-review of systematic reviews and meta-analyses, many face-to-face, nonpharmacological interventions are beneficial for caregivers [18]. This category of intervention includes psychoeducation, psychotherapy, occupational therapy, mindfulness interventions, and multicomponent interventions (eg, combining psychoeducation and psychotherapy components). Positive effects on depression, quality of life, sense of mastery, and communication skills have been attributed to various nonpharmacological interventions [18]. Although face-to-face interventions are effective, caregivers do not always use them and often find them difficult to access. This can be due to challenges such as being unable to leave the person alone, difficulty traveling, believing they do not need help, reluctance to seek services, limited knowledge of available resources, lack of time, or concerns about sharing personal information about their loved one [19].

To reduce some of these barriers, studies recommend that technological tools could make certain types of support more accessible to caregivers [20]. Among these, online interventions are effective and cost-effective and could favor accessibility [21,22]. A systematic review of online support interventions (eg, websites) for caregivers, including 12 studies [23], showed that these interventions can have positive effects on caregivers’ well-being. Among the interventions included in this systematic review, those that combine information, personalized strategies, and contact with other caregivers could increase caregivers’ knowledge and skills for caring for a loved one living with an MNCD, their confidence and self-efficacy, and reduce their stress, burden, and depressive symptoms [24-29]. Online interventions can easily incorporate a variety of formats (eg, videos, graphics, text), as well as support (eg, specific recommendations) [30]. The use of health information technologies can be relevant to family caregivers if adapted [31]. These technologies make it possible to reach isolated and stigmatized groups, reduce health care costs, and access information and advice at any time [32]. Among online support interventions, personalized ones (based on responses to a questionnaire or feedback following the intervention) show greater positive effects than nonpersonalized intervention [23,33,34].

In addition to online interventions, several technology applications (eg, sensors, wristwatches, cameras, voice recorders, calendars, automatic night lamps, gas cooker devices, picture button telephones, alarms, and electronic pillboxes) can support caregivers and people living with MNCDs [35]. In their scoping review, Huisman et al (2022) found that these technology applications help to reduce the burden and social isolation of caregivers and consequently promote better sleep. Technology applications for caregivers are mainly designed to provide information and resources, offer general practical tips for frequently encountered problems (eg, managing behavioral problems or medication), or help coordinate care among multiple caregivers (eg, sharing calendars or to-do lists) [36].

Although online interventions and technology applications have shown benefits and have many advantages when compared with face-to-face interventions, caregivers may find it difficult to personalize the content and translate the information provided into concrete strategies that fit specific everyday situations [37]. For example, the transposition of methods based on analysis of the situation encountered can represent a challenge [17]. Thus, to our knowledge, no tool that enables caregivers to learn how to use learning optimization methods to support the realization of meaningful activities by building on the person’s preserved abilities.

A previous study from our team highlighted the difficulty caregivers had in appropriating learning optimization methods, as well as their need for support tools to use these methods [38]. Based on this previous study, a knowledge mobilization study was carried out to codevelop a variety of support tools, including a web application, which is the scope of the present article. These support tools were designed to help caregivers learn, understand, and use learning optimization methods (eg, error-free learning). More precisely, the project aimed to support caregivers in adapting these methods to specific situations encountered daily and underlying difficulties to help people living with an MNCD maintain autonomy in meaningful activities by building on preserved abilities. This article describes the codevelopment and iterative steps involved in the design and validation process of a web application called “Aide-Mémoire-Interactif (AMI),” a support tool developed as part of this knowledge mobilization study.

## Methods

### Participatory Action Research Approach

To design the AMI web application, participatory action research based on a codevelopment and validation process was carried out. In the health care field, the codevelopment of such a web application requires a partnership among health care professionals, the people who will use the application, and the team of researchers and designers [39]. Caregivers, health and social service professionals, and workers from community organizations providing support to caregivers were involved in identifying situations frequently encountered by people living with an MNCD [16] and throughout the process of codeveloping and validating the AMI web application. The stages of this codevelopment were overseen by at least 1 coinvestigator with expertise in the stage in question and the principal investigator. For example, during the computer coding stage to operationalize the application, 2 project coinvestigators with expertise in this field supervised the computer programming work, and the principal investigator took part in all meetings with these coinvestigators and the computer scientist to ensure that user needs were met. The various stages leading to the codevelopment of the AMI web application can be grouped into 3 phases: (1) assessment of caregivers’ needs (identification of situations frequently encountered by people living with an MNCD) [16]; (2) production of appropriation tools, including the first version of the AMI web application [40]; and (3) validation of the tools, leading to the final version of the AMI application. Caregivers, health and social service professionals, and workers from community organizations experimented with the first version of the application during workshops with an occupational therapist and have made suggestions that contributed to revisions as the codevelopment progressed.

### Participants, Recruitment, and Data Collection

For the first stage of the project (ie, the identification of frequently encountered situations), 20 participants (caregivers, health and social service professionals, and community organization workers) were targeted to obtain a diversity of viewpoints and application contexts and data saturation [41]. Participants were recruited by one of the coinvestigators, a practitioner from the partner community organization. An interview guide for individual semistructured interviews (preplanned duration of approximately 60 min) was developed to identify meaningful activities and lived situations in which the cognitive difficulties of people living with an MNCD cause difficulties and would thus benefit from learning optimization methods. The results of this stage are reported in a previously published article [16].

For the 2 subsequent stages (ie, codevelopment and validation of the support tools), a target number of 8 to 10 participants was set for the learning optimization methods appropriation workshops. To ensure diversity of viewpoints in terms of application contexts while keeping the workshop group small enough to promote interactions, 3 participant groups were targeted: family caregivers, stakeholders from community organizations working with family caregivers, and occupational therapists in health and social services who work with people living with an MNCD in a variety of daily situations. A purposive sampling strategy was used to recruit women and men from different age groups and whether they were cohabiting or not with a person living with an MNCD. For family caregivers, inclusion criteria were (1) providing day-to-day support to a person living with an MNCD and (2) not having an MNCD as perceived by the referring caregiver. For occupational therapists and caregivers from community organizations, to meet the inclusion criteria, they had to work with people living with MNCDs and their caregivers. Occupational therapists were recruited by the head of the home care department based on their knowledge of home care issues. The coresearcher from the partner organization (Société Alzheimer des Maskoutains–Vallée des Patriotes) recruited community workers and caregivers by contacting caregivers who attended the organization’s activities and who, he felt, could contribute to the development of the appropriation tools by experimenting with and critiquing them.

### Production of Appropriation Tools

Several tools for appropriating learning methods were designed during the project, including workshops for caregivers [34], the AMI web application, companion leaflets, and video capsules showing examples of putting learning optimization methods into action, to support their operationalization in specific situations and contexts encountered. This study describes more specifically the codevelopment of the AMI application using a software development process based on the Agile Scrum methodology [42]. The Agile Scrum method is a project management methodology that follows an iterative process with users to adjust to changing objectives and needs. It is divided into short cycles, called “sprints,” which enable features to be developed progressively while adjusting priorities based on feedback from stakeholders (caregivers, occupational therapists, stakeholders from partner organizations, coresearchers, and research assistants). This methodology requires teams with the necessary complementary expertise and an operating structure based on defined roles to ensure communication and ongoing project progress. The design is therefore based on principles of user friendliness and accessibility to provide a practical and effective solution for specific contexts [43]. To promote accessibility, the application is accessed via a web browser, making it available on different devices and operating systems. Moreover, platform independence reduces development effort and costs involved in supporting multiple user installations. Finally, updates and the addition of new functionalities are possible, enabling the application to evolve in line with emerging needs.

The Agile Scrum methodology chosen was built around 4 elements targeted by the team. First, an ordered list of features for the AMI application was created based on the developed logic algorithm and usage requirements. Second, planning of the functionalities to be programmed was divided into subtasks per 2-week period. Third, daily meetings between the programmer and 2 coinvestigators (an occupational therapist and a computer scientist) were held to identify problems and find solutions, which were implemented by the programmer. Finally, demonstrations of developed functionalities and reviews of the latest iteration were carried out by the programmer and the 2 coinvestigators during the first workshop with caregivers and occupational therapists, enabling participants to experiment with the AMI application, criticize it, and validate the changes made as they experimented during the series of workshops.

### Tool Validation

**Figure 1.**
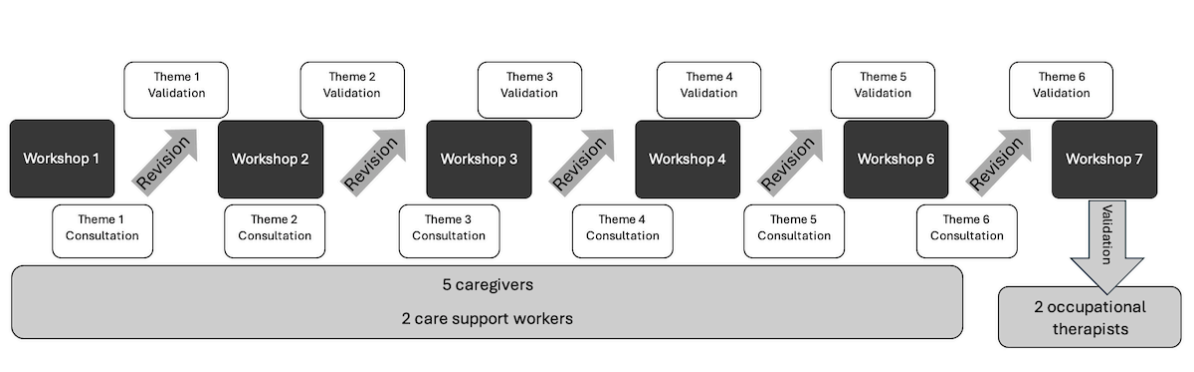
Codevelopment and validation process of the Aide-Mémoire-Interactif application.

A first version of the AMI web application was designed by the research team, followed by several feedback loops aimed at adjusting the application according to the comments received (Figure 1). Before the experimentation by caregivers, the application was tested by members of the research team and representatives of partner organizations. This allowed us to clarify some terms and add some answer choices. During the trial period, caregivers participating in the workshops provided feedback during demonstrations at each of the workshops and by trying out the application for themselves at home with their loved one afterwards [36]. After each workshop on a specific theme (eg, memory or executive function difficulties), feedback on the use of the AMI web application was collected, and modifications were made on an ongoing basis. For example, caregivers were asked to provide specific situations they wanted to be included in the app. For each situation, they answered the questions asked by the AMI web application and reviewed the suggested strategy. Participants were invited to provide feedback on various aspects of the app, including the variety of situations or suggestions for additional or missing ones, answer choices that could be missing for specific situations, cognitive difficulties encountered for a specific situation, and the actions included in the learning sequence. Modifications were then validated with participants during the following workshops, leading to the final version of the application after the workshop series. This approach, which included several iterations, made it possible to quickly adjust the development according to needs. Each caregiver had the opportunity to provide suggestions for the application based on specific situations they had encountered. The approach of valuing diversity and the richness of complementary or divergent points of view was presented right from the first workshop. It was specified that everyone’s experience was essential to the codevelopment of a relevant web application. Opposing comments were discussed as a group to identify a solution that would meet the needs of all participants. The principal researcher, who cofacilitated the workshops, took note of the participants’ comments and then discussed their implementation with the programmer and the information technology coresearcher.

### Ethical Considerations

This study was approved by the CIUSSS de l’Estrie-CHUS Research Ethics Committee (MP-31-2019-3211). Written informed consent was obtained from all participants. To ensure privacy, any information that could lead to identification was removed. Information gathered was kept confidential. No financial compensation was given to the participants.

## Results

### Process and Participants

This section describes the different steps that led to the development of the AMI web application and focuses on 2 complementary aspects: the participatory process through which the application was developed and the adjustments made to the tool based on users’ feedback. These elements illustrate how the final version of the application, which is described in detail, emerged through iterative refinement.

**Figure 2.**
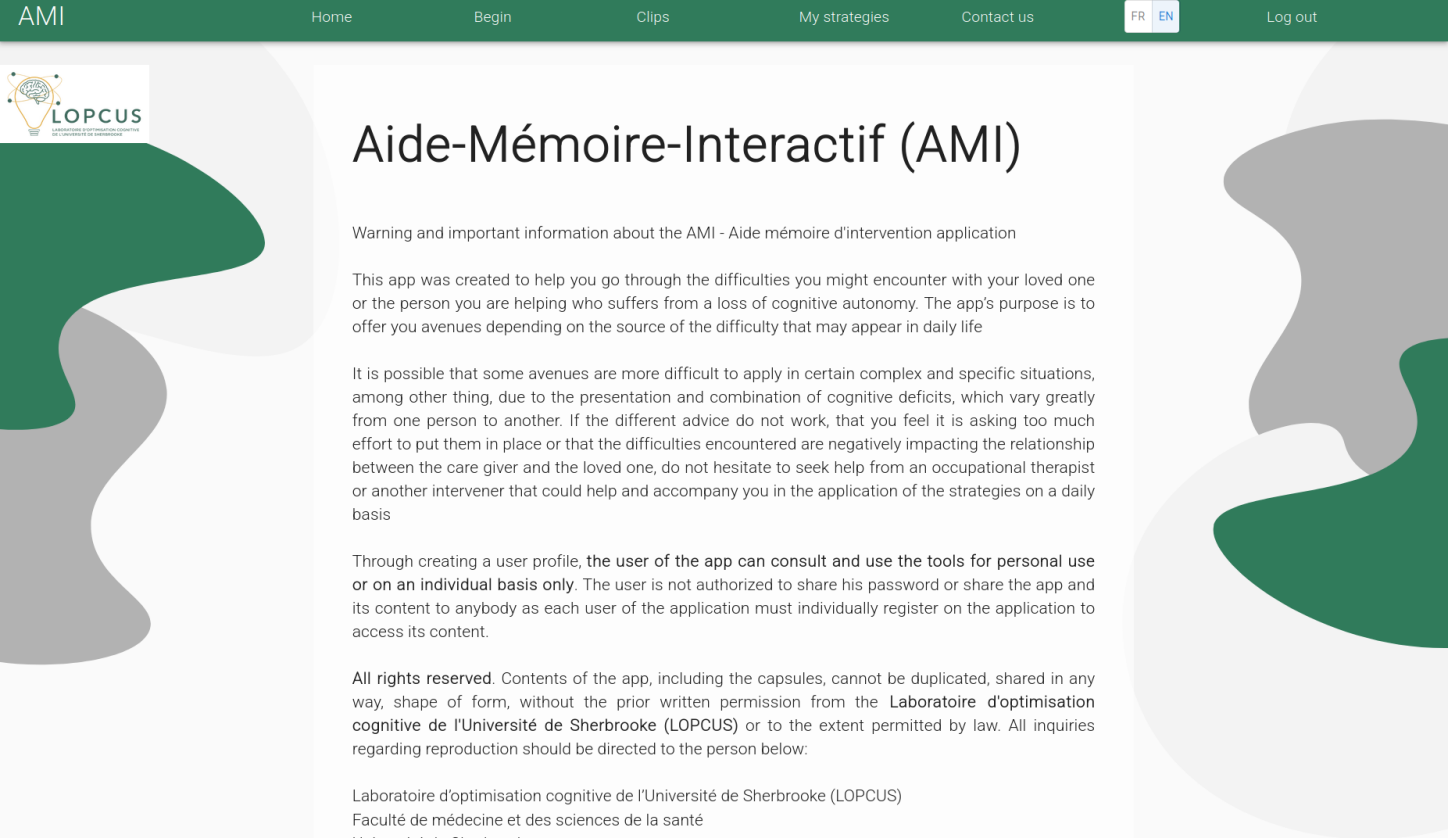
Aide-Mémoire-Interactif web application home page.

For the first part, caregivers (n=11), health and social service professionals (n=7; social workers and occupational therapists), and community organization workers (n=7) participated in identifying situations for which the AMI web application should support the operationalization of learning optimization methods. For the subsequent codevelopment and validation of the AMI web application, caregivers (n=5), caregiver support workers from community organizations (n=2), and occupational therapists working with people living with MNCDs (n=2) were recruited. The application was designed to be easy to navigate for users who are mainly caregivers or health care professionals working with people living with an MNCD [44]. The application home page (Figure 2) provides summary information on navigation, its various functionalities, and contact details for training in its optimal use. Indeed, the AMI web application, designed to support the operationalization of learning optimization methods, requires a basic understanding of the difficulties present in neurocognitive disorders and of these methods.

### Codevelopment of the AMI Web Application: Logical Architecture

Technological choices were made to ensure that the web application would be easy to adapt to the realities of partner environments. Thus, the logical architecture was built according to the rationale underlying learning optimization methods and is structured around the following questions: considering the specific situation in which the difficulty occurs, (1) What is the difficulty that can explain it? (2) What is the desired action? (3) After which action should the desired action be done? (4) What action is performed instead of the desired action? The application was codeveloped based on real-life situations requiring the use of learning optimization methods [39]. Question 1 asks the caregiver to identify, based on their perception, the primary cognitive difficulty that may explain why learning optimization methods are needed for this specific situation. The cognitive difficulty guides how the 4 learning optimization methods are combined to rely on preserved abilities. Question 2 requires the caregiver to analyze the sequence of actions, identify the usual error, and specify which action should be learned through motor encoding to replace this error. Question 3 also draws on the analysis of the action sequence and, in line with errorless learning principles, identifies the action that immediately precedes the error as well as the action that needs to be integrated using learning optimization methods. Finally, question 4 is essential to prevent the error before it occurs, supporting the errorless learning method. The strategy suggested by AMI includes a progression that integrates fading and spaced retrieval methods.

**Figure 3.**
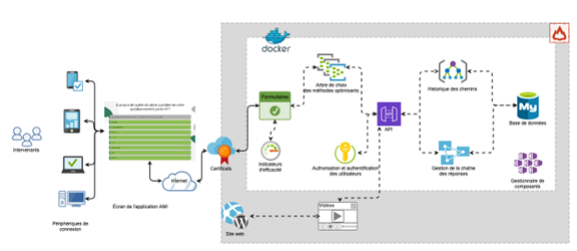
Logical architecture diagram of the Aide-Mémoire-Interactif web application.

Being user-centered, the AMI web application is built to offer each user dynamic interaction with the generated content so that the strategies generated are personalized to the situation encountered and the specific context. To optimize accessibility, the application is accessed using a web browser via a computer, tablet, or smartphone. As illustrated in Figure 3, AMI is organized around modular, adaptable software components comprising the following:

- a server secured by an SSL (Secure Sockets Layer) certificate, enabling access to data when the user interacts with the application, regardless of the device used;
- an API (application programming interface) that offers flexibility to integrate new components based on needs expressed by users during validation and scaling in different types of environments and with people with different levels of impairment;
- a Docker-type application virtualization environment, enabling the application to be used on any device;
- a MySQL database to store data for later access to customized strategies;
- a centralized Keycloak authentication and authorization component to facilitate user management [39] and ensure access to personalized strategies;
- an HTML interface for visualizing situation and context information entered by users;
- a ReactJS framework for rapid updating of suggested strategies based on user input;
- a high-performance, open-source FastAPI engine, enabling development, particularly with the Python language, to facilitate integration of new components and easily evolve the application in line with user needs.

The application is composed of scripts written in several programming languages. This plurality of programming languages allows greater modularity based on the requirements of each tool for programming learning methods. A 4-GB RAM server with 4 cores covers all application needs.

### Validation of the Tools: Perceptions and Feedback From Participants

As the workshops progressed, participants provided feedback that directly informed several modifications to the tool. Their suggestions mainly concerned the following: (1) formulation of everyday situations to make them easier to understand and choose from; (2) formulation of the difficulties underlying the problems in everyday situations to make them easier to understand and choose from; (3) addition of frequently encountered answer choices for desired actions and actions performed in place of desired actions; and (4) visual presentation for navigating questions, choices, and proposed strategies.

Following systematic verification of the vocabulary used in each situation category by participants, clarifications were made to facilitate understanding. Regarding the difficulties underlying the problems in everyday situations, certain categories of cognitive difficulties initially established were subdivided into 2 distinct categories to facilitate understanding and more direct application to the difficulties encountered. Regarding desired actions and actions performed in place of the desired action, additions were made to the answer choices to cover a wider range of contexts for operationalization in everyday situations. Additions were made to the list of answer choices, and if none of the options corresponded to the person’s situation, an “other” button led to an answer box for entering the context-specific element subsequently used by the AMI web application for personalized operationalization of learning optimization methods.

Some participants reported that certain dichotomous “yes or no” questions did not fit their situation, so they did not know which option to select. As a result, the “not applicable” option was added. Regarding the visual presentation of the AMI web application, feedback from participants helped to make navigation more intuitive (eg, replacing the “questionnaire” button with “start”). In response to participants’ request for the opportunity to save the strategies proposed for a specific situation, a “Favorites” button was created to save the individual history of strategies based on the individual answers provided to the questions. The AMI web application underwent refinements to support its ongoing improvement. Enhancements included the ability to save personalized strategies using the initials of both the caregiver and the care recipient. A printable version of the strategy, with editable sections to tailor intervention sequences, was also implemented. In parallel, visual and technical adjustments were made to optimize system performance, streamline navigation, and improve clarity for users. Participants confirmed that the latest version, shaped through iterative feedback, better met their needs.

### Final Version of the Application: Description of Functionalities

**Figure 4.**
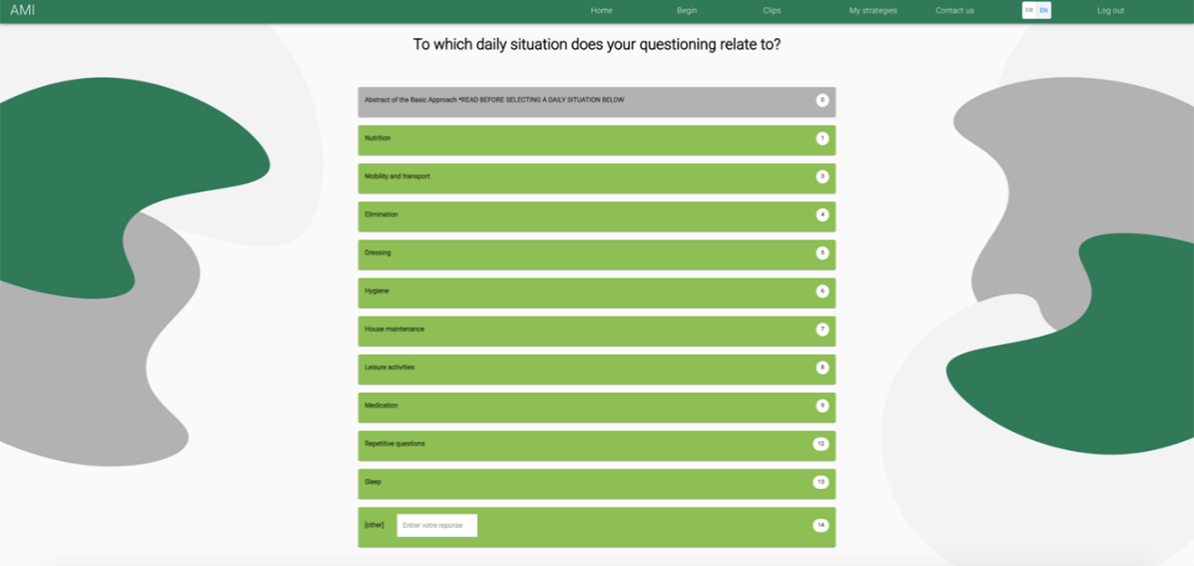
List of daily situations.

The home page of the final version of the AMI web application lets the user choose among the following: (1) starting navigation to operationalize learning optimization methods in a specific situation, (2) viewing video capsules describing these methods, or (3) consulting strategies saved from previous navigations. By selecting the Start option, users can select a daily situation from a range of possible options (Figure 4).

**Figure 5.**
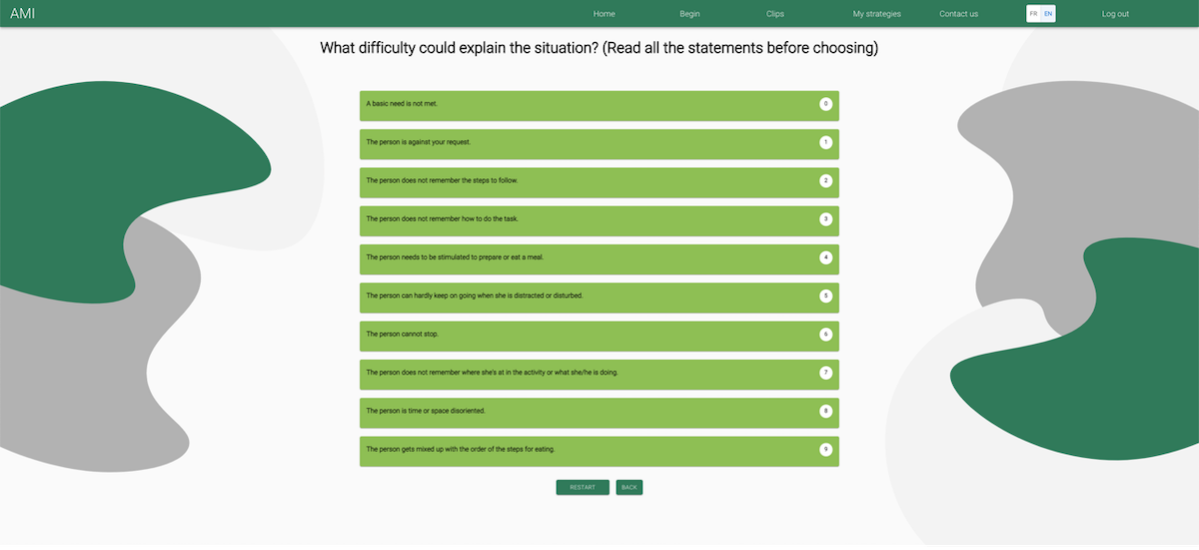
List of difficulties that could explain the situation.

Once a situation is selected, users identify the difficulty that could explain it. For example, for the Nutrition situation, 10 difficulties are presented (Figure 5). For each situation, questions and response options vary depending on the difficulty encountered.

**Figure 6.**
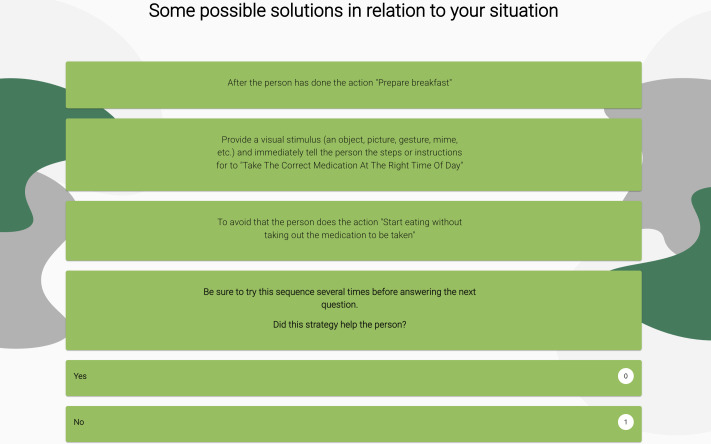
Example of a possible solution related to the situation.

Depending on the answers given about the situation (activity, cognitive difficulties) and the context in which it occurs, the user obtains suggestions for possible solutions to the situation (Figure 6).

Users can obtain a reminder of the theoretical foundations by clicking on Capsules. Twelve capsules are available, covering the following topics: (1) basic needs; (2) approaches (communication, validation, diversion, refusal); (3) methods to promote learning (error-free learning, motor encoding, spaced retrieval, blurring); (4) reminders of how memory, praxis, and executive functions work; (5) preserved abilities; and (6) complementarity with compensatory measures.

Finally, the *My Strategies* option in the drop-down menu includes 2 tabs: Lately, which allows the user to view the most recent sequences he or she has performed, and Favorites, which saves the operationalization of preferred strategies.

## Discussion

### Main Results

This participatory action research enabled us to codevelop and validate the AMI web application to support the operationalization of learning optimization methods for people living with an MNCD, by personalizing them to situations encountered daily as well as to the underlying cognitive difficulties. It enabled the reformulation of everyday situations and their underlying difficulties, the addition of frequently encountered answer choices for desired actions and actions performed in place of desired actions, and finally a visual presentation facilitating navigation. By involving caregivers [45], professionals, and users, this research contributed to the development of a web application that aligns with their needs and preferences.

### Comparison With Previous Studies

To date, a number of applications have been developed specifically for caregivers, with a variety of functions to meet the diverse needs of this population [20,31,32]. For its part, the AMI application is, to our knowledge, the first that facilitates the use of learning optimization methods by caregivers and that proposes solutions adapted to the specific context of everyday situations by promoting the use of the preserved abilities of people living with an MNCD. Indeed, when caregivers find themselves in a situation where they do not know how to deal with the impact of MNCDs on the performance of daily activities, the AMI web application provides them, after a series of questions on the specific context, with a strategy adapted to the problematic activity, the underlying cognitive difficulty, and the context in which it is performed [16]. Considering that several negative effects on physical and mental health can be caused by the role of caregiver, such as chronic stress and depression [46], feelings of burden [47], and poor-quality sleep [48], the possibility of having a web application that supports their role can help foster a positive aspect of the caregiver role.

The difficulties and activities performed by people living with MNCDs are highly variable, as are the coping skills of their caregivers, justifying the relevance and superior effectiveness of personalized interventions [23]. Warmoth et al [49] developed a personalized cognitive rehabilitation intervention and found that people living with MNCDs and their caregivers appreciated the personalized approach to intervention and that the flexibility allowed by this approach played an important role in their appropriation of learning optimization methods. Personalization is essential when using learning optimization methods, given the difficulty caregivers have in transposing these methods to another context [17]. The inclusion of users in studies aimed at developing services offered to them is essential [50] so that they can contribute to improving the products that meet their needs [51]. In the present study, the involvement of caregivers at all stages of the research enabled the application to be developed in line with the situations they encounter daily. In this way, the AMI application fills a gap for caregivers, enabling them to support the pursuit of day-to-day activities of people living with MNCDs.

Although the application is adapted to the situations encountered, studies report that lack of time and low digital literacy can limit the use of web applications by caregivers [52,53]. Since it can be difficult for some caregivers to find information [54], easy navigation and recording of information can be supportive. Clarity of language is also essential [52]. This participatory action research, which led to the codevelopment of the AMI application and involved seniors at every stage, enabled us to take these potential issues into account and make the necessary adjustments based on feedback from caregivers, mainly by adding the My Strategies feature to enable easy retrieval of personalized strategies.

Finally, studies tend to show that peer support and psychosocial support are also important elements in the interventions offered to caregivers [55]. Although the AMI web application was created to meet another equally important need of caregivers, the workshops held to help them appropriate the web application enabled several caregivers in similar situations to interact. This enabled them to share their experiences and possibly feel less alone, a feeling often experienced by people in this role [54]. In fact, 1 caregiver had recommended the addition of a function that would enable caregivers to obtain psychosocial support or communicate with other caregivers. Due to the extent of the research and programming required, which went beyond the primary objective of codevelopment, this recommendation could not be integrated into the current version of the application, despite its great relevance. These additional functionalities would be relevant given that the mobile web applications currently available for caregivers rarely address their personal needs, such as their health, financial security, or the problems they may encounter at work [56].

It would also fill a gap in web applications that currently do not offer support for caregivers’ emotional well-being, notably for stress and anxiety management, as well as depression [23-25,29].

### Strengths and Limitations

One of the main strengths of this study is the codevelopment process to ensure that the application meets needs and is user-friendly [37]. All types of potential users contributed to the codevelopment, including health care professionals and caregivers who worked in partnership with the team of researchers and designers (occupational therapy, social work, leisure, and information technology). Caregivers and health care professionals were involved throughout the process, from the definition of needs to the final validation of the application. Codevelopment ensures that the needs, preferences, and functionalities required by users are considered, and that the result is finally tailored to them [56]. Participants were caregivers and health care professionals mainly involved in home care. Although some have experience of intervention in a residential setting, the application answer choices are mainly based on situations occurring at home, in the community. Caregivers and interveners of people living with MNCDs in residential settings whose situations are not included in the application’s answer choices can, however, choose the *Other* option for the various questions and enter the contextual elements specific to their situation to obtain a personalized strategy proposal. These situations have not yet been validated. Although the number of caregivers involved was small, the multiple iterations allowed for the inclusion of a wide variety of situations. Future studies may adapt the application to other practice contexts (eg, hospital settings) or living environments (eg, residential resources offering long-term care) for people living with MNCDs. Although recruitment by reasoned choice targeted a diversity of experience, as caregivers and care providers were linked to a community organization and to health care network stakeholders working in collaboration with this organization, cultural representativeness and socioeconomic status were not present. More, since the caregivers were recruited targeting people who would be critical for a web application, since they had good digital literacy, they are not representative of all caregivers who may have more difficulty using technological tools. During implementation, introductory tools will have to consider the low digital literacy of some caregivers to guide their use of the web application. Although caregivers shared positive comments regarding the usefulness, accessibility, and effects of the AMI web application on autonomy and quality of the caregiving relationships, these effects were not assessed. Future studies should therefore assess the effects and usefulness of the AMI web application for caregivers and health professionals in supporting the performance of activities using the preserved abilities of people living with MNCDs. Future studies could evaluate the application’s implementation and its effects on caregivers, health professionals, and people living with an MNCD.

### Recommendations and Avenues of Research

The aim of this study was to describe the codevelopment process of the AMI web application, which supports the operationalization of learning optimization methods using the preserved abilities of people living with MNCDs. Suggestions received from caregivers and health care professionals have enabled us, in an iterative process, to improve the application so that it meets the specific needs of using preserved abilities in daily activities, thus compensating for difficulties encountered at home. Considering the physical and mental impacts of the caregiver role mentioned above, it would also be relevant to measure whether daily use of the AMI application has an impact on caregivers, particularly on their sense of burden. In the same way, the impact on the person living with an MNCD would also be interesting to assess. Second, it would also be relevant to look at the prolonged use of the AMI application to assess the sustainability of its use daily and to evaluate its long-term effectiveness [57], particularly in view of the progression of difficulties in daily activities attributable to the evolution of MNCD. Finally, as mentioned, additions to the web application would be relevant to make in future studies, such as offering peer support and psychosocial support via the application [53,58] and adding features that would support the personal needs of caregivers [55].

### Conclusions

This study details the steps that led to the development of the AMI web application, which enables caregivers to appropriate learning optimization methods for the specific situations encountered daily. The AMI application stands out for its ability to be customized to everyone’s context and for its codevelopment process, which involved caregivers, health care professionals, community workers, and researchers with expertise in the field. The AMI application was developed to provide a practical, high-performance solution for managing interventions. Its user-friendly interface, flexible web architecture, and advanced functionalities make it a valuable tool for professionals working in this specific field. What’s more, the technology used to develop the AMI web application has enabled optimal scalability and performance, guaranteeing a fluid user experience even with many simultaneous users. The chosen methodology favors the development of a web application that meets the needs of all those involved and thus optimizes its day-to-day use.

## Abbreviations

AMI: Aide-Mémoire-Interactif
MNCD: major neurocognitive disorder
